# Outlines of the Arteries and Nerves

**Published:** 1846-03

**Authors:** 


*Outlines of the Arteries and Nerves,* with short descriptions. By JOHN NEILL, M. D., Demonstrator of Anatomy in the University of Pennsylvania, &c. &c. Philadelphia: BARRINGTON & HASWELL, 1845.

These two little works, containing colored plates of the different parts of the arterial and nervous systems, will serve to refresh the mind of the student, after the study of more extensive works, with dissections, upon the subject. They are neatly gotten up, as is, indeed, almost every work brought out by the enterprising publishers.

